# The draft genome sequence of *Bacillus sp*. 5w2, a potential PHA producer, isolated from the bottom sediments of the Chornobyl nuclear power plant cooling pond

**DOI:** 10.1128/mra.00282-26

**Published:** 2026-05-18

**Authors:** Alina Kharchuk, Maksym Kharchuk, Maksym Kharkhota, Larysa Mozhaieva, Liliia Avdieieva

**Affiliations:** 1 D.K. Zabolotny Institute of Microbiology and Virology of the NASU, Kyiv, Ukraine; Fluxus Inc., Sunnyvale, California, USA

**Keywords:** *Bacillus*, polyhydroxyalkanoates (PHA), Chornobyl NPP, whole-genome sequencing

## Abstract

We present the draft genome of *Bacillus sp*. 5w2 containing 43 scaffolds with a total genome size of 5.78 Mb and a GC content of 35.5%. The strain is predicted to carry *pha* loci for the biosynthesis of polyhydroxyalkanoates.

## ANNOUNCEMENT

Members of the *Bacillaceae* family are ubiquitous microorganisms, famous for their remarkable stress resistance and production of industrially important secondary metabolites (1). *Bacillus* species are persistent inhabitants of contaminated ecosystems, including radionuclide-impacted areas like the Chornobyl exclusion zone (2). However, genomic data for *Bacillus* isolates recovered from the Chornobyl nuclear power plant (NPP) territory remain limited.

Here, we report the draft genome sequence of *Bacillus sp*. 5w2, isolated in 2018 from the bottom sediments of the Chornobyl NPP cooling pond in Ukraine (3). Aerobic spore-forming bacteria were isolated using serial dilution and heat treatment (80°C for 10–15 min) followed by cultivation on Luria–Bertani (LB) agar at 37°C for 48 h. Large, rough, dry, and adherent colonies typical of aerobic spore-formers were selected. For DNA isolation, the strain was cultured on LB agar at 37°C for 24 h. Genomic DNA was extracted using the Quick-DNA Miniprep Plus Kit (Zymo Research). Genomic DNA quantity was measured fluorometrically using the DeNovix dsDNA High-Sensitivity Assay Kit on a DeNovix DS-11 FX + Spectrophotometer/Fluorometer (DeNovix Inc., Wilmington, DE, USA); purity was assessed spectrophotometrically, and integrity was verified by agarose gel electrophoresis. DNA libraries were prepared using the MGIEasy Fast FS DNA Library Prep Set (Cat. No. 1000006987, MGI Tech). Sequencing was performed on the DNBSEQ PE150 platform with 100× genome coverage. A total of 1,239 Mb of sequence data and 8,259,176 reads were generated. Raw paired-end reads were quality-assessed using FastQC v0.12.1 (4). Adapter sequences and low-quality bases were trimmed using fastp v0.23.2 (5). The assembly was processed with Newbler v3.0 and quality-assessed using CheckM v1.2.4 (6). The final assembly comprised 43 scaffolds (53 contigs) totaling 5,781,623 bp, with a GC content of 35.5%, a scaffold N50 of 206.2 kb, and a contig N50 of 186.3 kb. The level of completeness was 98.24% with 0.25% contamination, according to the CheckM analysis. Genome annotation was performed using NCBI PGAP (v. 6.10) (7), predicting 6,051 genes, including 5,769 protein-coding genes and 81 RNA genes, including three 5S rRNAs, one 16S rRNA, one 23S rRNA, 71 tRNAs, and 5 ncRNAs. Prokka (8) identified 5,899 coding sequences. EggNOG-mapper (9) assigned Gene Ontology terms and KEGG Pathways groups to 5,456 of the predicted proteins (92.47%). Taxonomic classification using GTDB-Tk (release R226) (10) placed strain 5w2 within the genus *Bacillus*. ANI analysis revealed 99.08% identity to the reference genome *Bacillus sp*. dmp10 (GCF_003400245.1), while no species-level reference genome exceeded the 96% ANI delineation threshold, with the highest similarity to *B. wiedmannii* GCF_001583695.1 genome (ANI 95.09%, AF 0.837).

Genome mining using antiSMASH v8.0.4 (11) identified biosynthetic gene clusters for a paeninodin-like lassopeptide (MIBiG BGC0001356), a bacillibactin siderophore (MIBiG BGC0000309), petrobactin (MIBiG BGC0000942), and pulcherriminic acid (MIBiG BGC0002103). Additionally, a complete and conserved PHA biosynthetic gene cluster was identified, possibly responsible for polyhydroxyalkanoate production.

## Data Availability

All data are available under BioProject PRJNA1332261, BioSample SAMN52661587, SRA SRX32392185, and Assembly GCA_054353775.1. Manual annotations are available on Zenodo: https://doi.org/10.5281/zenodo.18875413.

## References

[1] Herrmann LW, Letti LAJ, Penha R de O, Soccol VT, Rodrigues C, Soccol CR. 2024. Bacillus genus industrial applications and innovation: first steps towards a circular bioeconomy. Biotechnol Adv 70:108300. doi:10.1016/j.biotechadv.2023.108300 38101553

[2] Romanovskaya VA, Rokitko PV, Mikheev AN, Gushcha NI, Malashenko YuR, Chernaya NA. 2002. The effect of γ-radiation and desiccation on the viability of the soil bacteria isolated from the alienated zone around the chernobyl nuclear power plant. Microbiology (Reading, Engl) 71:608–613. doi:10.1023/A:1020575223365 12449639

[3] Kharkhota M, Hrabova H, Kharchuk M, Ivanytsia T, Mozhaieva L, Poliakova A, Avdieieva L. 2022. Chromogenicity of aerobic spore-forming bacteria of the Bacillaceae family isolated from different ecological niches and physiographic zones. Braz J Microbiol 53:1395–1408. doi:10.1007/s42770-022-00755-9 35438476 PMC9433553

[4] Andrews S. 2010. FastQC: A quality control tool for high throughput sequence data. Babraham Bioinformatics. https://www.bioinformatics.babraham.ac.uk/projects/fastqc/.

[5] Chen S, Zhou Y, Chen Y, Gu J. 2018. Fastp: an ultra-fast all-in-one FASTQ preprocessor. Bioinformatics 34:i884–i890. doi:10.1093/bioinformatics/bty560 30423086 PMC6129281

[6] Parks DH, Imelfort M, Skennerton CT, Hugenholtz P, Tyson GW. 2015. CheckM: assessing the quality of microbial genomes recovered from isolates, single cells, and metagenomes. Genome Res 25:1043–1055. doi:10.1101/gr.186072.114 25977477 PMC4484387

[7] Tatusova T, DiCuccio M, Badretdin A, Chetvernin V, Nawrocki EP, Zaslavsky L, Lomsadze A, Pruitt KD, Borodovsky M, Ostell J. 2016. NCBI prokaryotic genome annotation pipeline. Nucleic Acids Res 44:6614–6624. doi:10.1093/nar/gkw569 27342282 PMC5001611

[8] Seemann T. 2014. Prokka: rapid prokaryotic genome annotation. Bioinformatics 30:2068–2069. doi:10.1093/bioinformatics/btu153 24642063

[9] Cantalapiedra CP, Hernández-Plaza A, Letunic I, Bork P, Huerta-Cepas J. 2021. eggNOG-mapper V2: functional annotation, orthology assignments, and domain prediction at the metagenomic scale. bioRxiv. doi:10.1101/2021.06.03.446934 PMC8662613 34597405

[10] Chaumeil P-A, Mussig AJ, Hugenholtz P, Parks DH. 2022. GTDB-TK V2: memory friendly classification with the genome taxonomy database. Bioinformatics 38:5315–5316. doi:10.1093/bioinformatics/btac672 36218463 PMC9710552

[11] Blin K, Shaw S, Vader L, Szenei J, Reitz ZL, Augustijn HE, Cediel-Becerra JDD, de Crécy-Lagard V, Koetsier RA, Williams SE, et al.. 2025. antiSMASH 8.0: extended gene cluster detection capabilities and analyses of chemistry, enzymology, and regulation. Nucleic Acids Res 53:W32–W38. doi:10.1093/nar/gkaf334 40276974 PMC12230676

